# Mapping Potential Determinants of Peroxidative Activity in an Evolved Fungal Peroxygenase from *Agrocybe aegerita*

**DOI:** 10.3389/fbioe.2021.741282

**Published:** 2021-09-14

**Authors:** Patricia Molina-Espeja, Alejandro Beltran-Nogal, Maria Alejandra Alfuzzi, Victor Guallar, Miguel Alcalde

**Affiliations:** ^1^ Department of Biocatalysis, Institute of Catalysis, CSIC, Madrid, Spain; ^2^ Barcelona Supercomputing Center, Barcelona, Spain; ^3^ ICREA, Institució Catalana de Recerca i Estudis Avançats Passeig Lluís Companys, Barcelona, Spain

**Keywords:** Fungal unspecific peroxygenase, peroxidative activity, peroxygenative activity, long range electron transfer pathway, heme access channel, directed evolution

## Abstract

Fungal unspecific peroxygenases (UPOs) are hybrid biocatalysts with peroxygenative activity that insert oxygen into non-activated compounds, while also possessing convergent peroxidative activity for one electron oxidation reactions. In several ligninolytic peroxidases, the site of peroxidative activity is associated with an oxidizable aromatic residue at the protein surface that connects to the buried heme domain through a long-range electron transfer (LRET) pathway. However, the peroxidative activity of these enzymes may also be initiated at the heme access channel. In this study, we examined the origin of the peroxidative activity of UPOs using an evolved secretion variant (PaDa-I mutant) from *Agrocybe aegerita* as our point of departure. After analyzing potential radical-forming aromatic residues at the PaDa-I surface by QM/MM, independent saturation mutagenesis libraries of Trp24, Tyr47, Tyr79, Tyr151, Tyr265, Tyr281, Tyr293 and Tyr325 were constructed and screened with both peroxidative and peroxygenative substrates. These mutant libraries were mostly inactive, with only a few functional clones detected, none of these showing marked differences in the peroxygenative and peroxidative activities. By contrast, when the flexible Gly314-Gly318 loop that is found at the outer entrance to the heme channel was subjected to combinatorial saturation mutagenesis and computational analysis, mutants with improved kinetics and a shift in the pH activity profile for peroxidative substrates were found, while they retained their kinetic values for peroxygenative substrates. This striking change was accompanied by a 4.5°C enhancement in kinetic thermostability despite the variants carried up to four consecutive mutations. Taken together, our study proves that the origin of the peroxidative activity in UPOs, unlike other ligninolytic peroxidases described to date, is not dependent on a LRET route from oxidizable residues at the protein surface, but rather it seems to be exclusively located at the heme access channel.

## Introduction

Fungal unspecific peroxygenases (UPOs, EC 1.11.2.1) belong to a unique group of heme-thiolate peroxidases that catalyze the insertion of oxygen into non-activated C-H bonds through their peroxygenative activity (a two electron oxidation route) (Hofrichter et al., 2020). With a substrate range of more than 300 compounds, this promiscuous biocatalyst is simply triggered by H_2_O_2_ to perform a variety of oxyfunctionalization reactions, including aromatic and aliphatic hydroxylations, aromatic and aliphatic epoxidations, sulfoxidations, *N*-oxidations, *N*-dealkylations, brominations and ether cleavage (Bormann et al., 2015; Hofrichter and Ullrich, 2014; Münch et al., 2021; Sigmund and Poelarends, 2020), Figure 1. More than 4,000 putative UPO sequences have been deposited in the genome databases, representing a large source of natural UPOs [from both the long and short families (Hofrichter et al., 2020)] that can be used for future enzyme engineering endeavors and practical applications, such as for the production of pharmaceuticals, fine and bulk chemicals, in environmental bioremediation and beyond (Grogan, 2021 and reference herein). However, there are several issues that must be addressed before UPOs can be considered a natural replacement for the long-in-the-tooth P450 monooxygenases. Among these are their functional expression in appropriate industrial hosts, their oxidative inactivation by H_2_O_2_ and the coexistence of two activities within the same protein scaffold: peroxygenative and peroxidative (Hofrichter et al., 2015; Holtmann and Hollmann, 2016; Wang et al., 2017). While the first two problems have been studied extensively and virtually resolved (Hobisch et al., 2020 and references herein), the convergence of peroxygenative activity (an oxygen transfer reaction by two electron oxidation with peroxide as an oxygen source) with peroxidative activity (one electron oxidation) make UPOs unsuitable for several industrial applications (Molina-Espeja et al., 2016; Molina-Espeja et al., 2017), Figure 1. For instance, this is an important concern in the pharmaceutical industry for the selective hydroxylation of aromatic compounds, such as in the production of active pharmaceutical ingredients (APIs) to that of human drug metabolites that requires very pure final hydroxylated products to be generated in large amounts (Kiebist et al., 2019). Given that the resulting phenolic product of the peroxygenative activity on aromatic compounds may be substrates of the peroxidative activity of these enzymes, a pool of oxidation by-products (phenoxyl radicals and quinones) is likely to be released along the way, promoting further non-enzymatic polymerizations that diminish the production yields and complicate downstream processing (Gomez de Santos et al., 2018). As such, it is important to understand the catalytic determinants behind the generally undesired peroxidative activity of UPOs.

**FIGURE 1:**
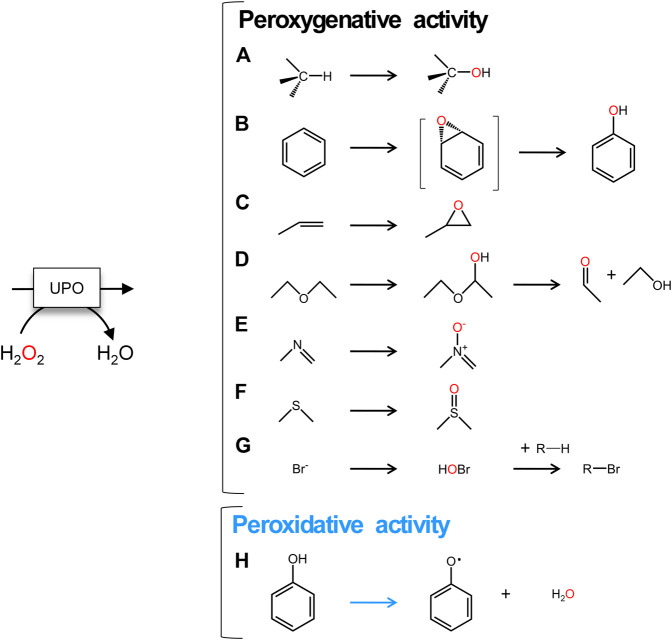
UPO reactions. **A** to **G**, two electron oxidations: **(A)** aliphatic hydroxylation; **(B)** aromatic hydroxylation; **(C)** epoxidation; **(D)** ether cleavage; **(E)** *N*-oxidation; **(F)** *S*-oxidation; **(G)** halogenation. **(H)** One electron oxidation of aromatic compounds. Peroxygenative reactions are shown with black arrows and peroxidative reaction is indicated with a blue arrow.

In fungal ligninolytic peroxidases, the catalytic binding site for peroxidative substrates is located at either the heme access channel or the protein surface, the latter involving a catalytic aromatic residue (Trp or Tyr) that is exposed to the solvent and that transfers one electron upon substrate oxidation to the heme through a long-range electron transfer (LRET) pathway (Ruiz-Dueñas et al., 2008). Indeed, such surface oxidizable Trp/Tyr residues act as active catalytic radicals in LRET pathways and are involved in the oxidation of high-redox potential compounds that cannot take place directly at the heme site, such as that of veratryl alcohol or recalcitrant dyes. For instance, surface Trp residues are thought to drive LRET in the lignin peroxidase (LiP) from *Phanerochaete chrysosporium* (Doyle et al., 1998) and the versatile peroxidase (VP) from *Pleurotus eryngii* (Perez-Boada et al., 2005), and more recently a mixed tryptophanyl/tyrosyl radical was identified in the dye-decolorizing peroxidase (DyP) from *Auricularia auricula-judae* (Linde et al., 2015). Conversely, the heme access channel has been long described as the main oxidation site for low-redox potential compounds in generic peroxidases, although VP oxidizes low-redox potential compounds at both the heme access channel (with low efficiency) and at the catalytic surface Trp (with high efficiency (Ruiz-Dueñas et al., 2008)).

Through directed evolution, we previously designed an UPO mutant (referred to as PaDa-I) that was derived from the *Cyclocybe* (*Agrocybe*) *aegerita* UPO (*Aae*UPO). This evolved UPO variant was functionally expressed in yeast in a highly active and soluble form, at high secretion titers, and it has been used widely as a model enzyme in several UPO engineering studies (Molina-Espeja et al., 2014; Molina-Espeja et al., 2015). Recently, we resolved the crystal structure of PaDa-I in the presence of an array of substrates, revealing key features of the promiscuous oxygen transfer activity of this enzyme and in particular, highlighting the different conformational states of the flexible Gly314-Gly218 loop at the heme access channel upon substrate binding (Ramirez-Escudero et al., 2018). Here, we have examined potential determinants of the PaDa-I mutant’s peroxidative activity. After performing a quantum mechanics/molecular mechanics (QM/MM) analysis, eight potential radical-forming aromatic residues at the protein’s surface were studied by saturation mutagenesis, which along with the construction of a combinatorial saturation mutagenesis library targeting loop Gly314-Gly318 followed by a substrate diffusion computational analysis, allowed us to shed light on the determinants of the peroxidative activity in this UPO.

## Materials and Methods

### Materials

PaDa-I is the parental type used in this work and was obtained as described elsewhere (Molina-Espeja et al., 2014). Expression shuttle vector pJRoC30 containing uracil auxotrophy and ampicillin markers for selection came from California Institute of Technology (CALTECH, United States). NBD (5-nitro-1,3-benzodioxole) was purchased from TCI America (Tokio, Japan). ABTS [2,2′-azino-bis-(3-ethylbenzothiazoline-6-sulfonic acid)] and Yeast transformation kit were purchased from Sigma-Aldrich/Merck (Darmstadt, Germany) and the high-fidelity DNA polymerase iProof from Bio-Rad (Hercules, CA, United States). *Escherichia coli* XL1-Blue competent cells were purchased at Agilent Technologies (Santa Clara, CA, United States) while *Saccharomyces cerevisiae* strain BJ5464 was from LGC Promochem (Barcelona, Spain). Zymoprep yeast plasmid miniprep kit was purchased to Zymo Research (Orange, CA, United States) and NucleoSpin Plasmid kit was from Macherey-Nagel (Düren, Germany). Restriction enzymes BamHI and XhoI were purchased from New England Biolabs (Hertfordshire, United Kingdom). All chemicals were reagent-grade purity.

### Culture Media

Minimal medium, SC drop-out plates and Luria-Bertani (LB) medium were prepared as reported elsewhere (Mate et al., 2017). Selective expression medium (SEM) contained 100 ml 67 g/L filtered yeast nitrogen base, 100 ml 19.2 g/L filtered yeast synthetic drop-out medium without uracil, 100 ml 20% filtered galactose, 67 ml 1 M filtered KH_2_PO_4_ pH 6.0 buffer, 22 ml 0.1 M filtered MgSO_4_, 34.8 ml absolute ethanol, 1 ml 25 g/L filtered chloramphenicol and _dd_H_2_O up to 1 L. Expression medium included 712.5 ml 1.55X YP, 66 ml 1 M filtered KH_2_PO_4_ pH 6.0 buffer, 110 ml 20% filtered galactose, 22 ml 0.1 M filtered MgSO_4_, 31.5 ml absolute ethanol, 1.1 ml 25 g/L filtered chloramphenicol and _dd_H_2_O up to 1 L.

### Computational Analysis

The starting model for the QM/MM and PELE simulations was prepared from the 5OXU PaDa-I crystal structure. Using the protein preparation wizard from Schrodinger, we prepared the protein structure at pH 7.0 (optimal peroxygenase activity pH) and 4.5 (optimal peroxidase activity pH). The heme site was modeled as compound I after being fully optimized in the protein environment with a QM/MM optimization using Qsite from Schrtodinger. The same software was used then for all QM/MM spin density calculations. QM/MM simulations included all tyrosine (Tyr47, Tyr79, Tyr151, Tyr160, Tyr194, Tyr265, Tyr281, Tyr293 and Tyr325) and tryptophan (Trp24) residues in the quantum region (Supplementary Figure 1), subtracting one electron and computing spin density. Thus, QM/MM results were analyzed in terms of spin density populations (appearance of radical character), rather than energetic or structural analysis.

PELE simulations, aimed at describing ABTS diffusion and binding, used the model prepared at pH 4.5. PELE is a molecular mechanics Monte Carlo (MC) software capable of efficiently describing the non-biased binding site search and induced fit (Acebes et al., 2016). The pocket search workflow was used, using 128 trajectories (computing cores) where each one is initiated placing randomly the substrate along the enzyme surface and proceeds with an adaptive PELE simulation, using 20 epochs of 25 MC each (Lecina et al., 2017). Each enzyme-substrate pose was then characterized by: 1) the distance from the substrate center of mass to Ala316 beta carbon, 2) the enzyme-substrate interaction energy computed at the OPLS-AA force field and a generalized Born solvent.

### Saturation and Combinatorial Saturation Mutagenesis Libraries

Trp24, Tyr47, Tyr79, Tyr151, Tyr265, Tyr281, Tyr293 and Tyr325 were subjected to site saturation mutagenesis whereas Gly314, Val315, Ala316, Ala317, Gly318 (loop Gly314-Gly318) were targeted to combinatorial saturation mutagenesis (CSM). Two high-fidelity PCRs were performed in a final volume of 50 µL containing: 3% DMSO, 1 mM dNTPs (0.25 mM each), 0.02/µL iProof DNA polymerase, 0.2 ng/μL template (PaDa-I), and: 1) 0.5 µM RMLC and 0.5 µM DIRSAT 2) 0.5 µM RMLN and 0.5 µM REVSAT. The following PCR parameters were used for each reaction: 1) 98°C for 30 s (1 cycle), 98°C for 10 s, 52°C for 25 s, 72°C for 15 s (28 cycles) and 72°C for 10 min (1 cycle); 2) 98°C for 30 s (1 cycle), 98°C for 10 s, 47°C for 25 s, 72°C for 45 s (28 cycles) and 72°C for 10 min (1 cycle). The sequences of all primers are shown in Supplementary Table 1. PCR products were loaded onto a preparative agarose gel and isolated using the Zymoclean Gel DNA Recovery kit. The recovered PCR products (200 ng each) were mixed with the linearized vector (100 ng) and transformed into competent *S. cerevisiae* cells using the Yeast Transformation kit for *in vivo* gene re-assembly and cloning. The DNA fragments were cloned under the control of the GAL1 promoter of the pJRoC30 expression shuttle vector, using BamHI and XhoI to linearize the plasmid. *In vivo* ligation was promoted by designing ∼50 bp overhangs with homology of sequence to the linear vector ends. Transformed cells were incubated for 3 days at 30°C on minimum synthetic drop-out plates.

### High-Throughput Screening

Selected colonies were cultured in sterile 96-well plates containing 200 µL of expression medium per well. In each plate, one well (H1) was not inoculated to serve as a negative control and column 6 was inoculated with parental PaDa-I. Plates were incubated at 30°C, 220 rpm and 80% relative humidity in a shaker (Minitron-INFORS, Switzerland) for 3 days. Afterwards, plates were centrifuged (Eppendorf 5810R centrifuge, Germany) for 10 min, 2,500 rpm and 4°C. A robotic liquid handling station (Freedom EVO 100 base, TECAN Schweiz AG, Switzerland) transferred supernatant aliquots (20 µL) to new replica plates. With the help of a pipetting robot (Multidrop Combi Reagent Dispenser, Thermo Scientific), 180 µL of reaction mixtures with ABTS or NBD were added to each replica plate. ABTS reaction mixture contained 0.3 mM ABTS, 100 mM sodium phosphate-citrate buffer pH 4.0 and 2 mM H_2_O_2_. NBD reaction mixture contained 1 mM NBD (dissolved in 100% acetonitrile, ACN, with a final concentration of 15% ACN in each well), 100 mM potassium phosphate buffer pH 7.0 and 1 mM H_2_O_2_. The plates were stirred and the absorbance was measured using a plate reader (SPECTRAMax Plus 384, Molecular Devices, Sunnyvale, CA, United States) in kinetic mode at 418 nm (*ε*_ABTS_ = 36,000 M^−1^.cm^−1^) for ABTS and in end point mode at 425 nm (*ε*_NBD_ = 9,700 M^−1^.cm^−1^) for NBD. The values were normalized against the corresponding parental in each plate. 20–30 clones were selected to be tested again in a first re-screening.

First re-screening: The selected clones and the parental type PaDa-I were inoculated in sterile 96-well plates containing 200 µL of expression medium per well and cultured as described above. Each clone was grown in five consecutive wells to serve as replicates. Columns 1 and 12 and rows A and H were excluded to avoid potential false positives caused by evaporation. Clones were screened following the screening protocol described above. Seven clones with remarkable activities were selected for second rescreening.

Second rescreening: The selected clones (100 µL) were grown in 3 ml of YPD medium at 30°C and 220 rpm for 24 h. Plasmids were extracted from aliquots of 1.5 ml and used to transform *E. coli* XL-1 Blue cells. Cells were spread in LB plates and incubated overnight at 37°C. Individual colonies were picked and grown in 5 ml of LB liquid medium. Plasmids were extracted using the Nucleospin Plasmid kit and the product was used to transform competent *S. cerevisiae* cells. The transformed cells were plated in minimum selective synthetic drop-out plates and incubated for 3 days at 30°C. Single colonies were then inoculated in 3 ml of minimum medium and incubated for 3 days at 30°C and 220 rpm. Clones were refreshed reaching an optical density OD_600_ = 0.25 in a final volume of 3 ml minimum medium. The cultures were subjected to two cycles of growth (OD_600_ ∼ 1) and then used to inoculate rich expression medium to a final volume of 10 ml in order to get a final OD_600_ = 0.1. The cultures were incubated for 3 days at 25°C and 220 rpm to induce protein expression. Cultures then were centrifuged at 5,000 rpm and 4°C for 10 min to remove the cells from the supernatant. The supernatant of each clone and the parental type were subjected to the screening assays for ABTS and NBD as described previously. Appropriate dilutions of supernatants were prepared in such a way that aliquots of 20 μL gave rise to a linear response in kinetic mode.

### Protein Purification

Clones 8, 23 and PaDa-I were produced and purified to homogeneity. A single *S. cerevisiae* colony from each variant was inoculated in 20 ml of minimal medium and incubated for 48 h at 30°C and 220 rpm. Clones were refreshed in a final volume of 100 ml minimal medium at an optical density OD_600_ = 0.3. After 6–8 h of growing (OD_600_ = 1–1.5), 720 ml of expression medium were inoculated with 80 ml pre-culture and grown 72 h at 25°C and 120 rpm. Cells were removed by centrifugation at 6,000 rpm and 4°C during 30 min saving the supernatants for enzyme assays. Supernatants were filtered using a nitrocellulose membrane of 0.45 µm pore size. Then, supernatants were concentrated using a Pellicon tangential ultrafiltration system (10 kDa cut-off membrane; Millipore, United States) and an Amicon stirred ultrafiltration cell (10 kDa cut-off membrane; Millipore, United States), followed by dialysis against 20 mM sodium phosphate-citrate pH 3.3 buffer (buffer A). The samples were filtered and loaded into one cation-exchange HiTrap SP FF column connected to an ÄKTA purifier system (GE Healthcare, United Kingdom) and pre-equilibrated with buffer A. The proteins were eluted with a linear gradient from 0 to 40% in 40 min of buffer A containing 1 M NaCl. Fractions with ABTS activity were collected, concentrated and dialyzed against 20 mM Tris-HCl pH 7.8 buffer (buffer B) and loaded into a HiTrap Q FF anion-exchange column (GE Healthcare, United Kingdom), pre-equilibrated with buffer B. Proteins were eluted with a linear gradient from 0 to 20% in 30 min of buffer B containing 1 M NaCl. The fractions with UPO activity towards ABTS were collected and dialyzed against 20 mM potassium phosphate pH 7.0 buffer. Samples of pure enzymes were stored at 4°C. The Reinheitszahl values [R_Z_] [A_418_/A_280_] achieved were ∼2. Throughout the purification protocol, the fractions were analysed by SDS/PAGE on 12% gels and the proteins were stained with SeeBand Protein Staining solution (Gene Bio-Application Ltd., Israel). The concentrations of all crude protein extracts were determined using the Bio-Rad protein reagent and BSA as standard.

### Biochemical Characterization

Kinetic parameters: Kinetic values were estimated with increasing substrate concentrations and fitted to a single rectangular hyperbola function of the Michaelis-Menten model by using the Enzyme Kinetics module of SigmaPlot 12.0. Kinetics for ABTS were measured in 100 mM sodium phospate-citrate buffer pH 4.0 (for PaDa-I) or pH 3.0 (for clones 8 and 23) containing 2 mM of H_2_O_2_. Kinetics for NBD were performed in 100 mM potassium phosphate buffer pH 7.0 containing 1 mM of H_2_O_2_ in 15% of acetonitrile. Kinetics for veratryl alcohol were carried out in 100 mM potassium phosphate buffer pH 7.0 containing 2 mM of H_2_O_2._ For each substrate, reactions were performed by triplicate following the increase of the absorption for ABTS^•+^ (*ε*_418_ = 36,000 M^−1^.cm^−1^); NBD (*ε*_425_ = 9,700 M^−1^.cm^−1^); and veratryl alcohol (*ε*_310_ = 9,300 M^−1^.cm^−1^).

pH activity profiles: Appropriate enzyme dilutions were prepared in such a way that 20 µL of enzyme dilution produced a linear response in the kinetic mode. Reaction mixtures were prepared at pH values 2.0, 3.0, 4.0, 5.0, 6.0, 7.0, 8.0 and 9.0 in 100 mM citrate/phosphate/borate buffer with 2 mM H_2_O_2_ and 2 mM ABTS. The assay commenced when 180 µL of reaction mixture were added to each well containing 20 µL of enzyme dilution. The activities were measured in triplicate in kinetic mode by following the increase of the absorption for ABTS^•+^ (*ε*_418_ = 36,000 M^−1^.cm^−1^) and the relative activity (in percent) is based on the maximum activity for each variant in the assay.

Thermostability assay (*T*_50_): 50 µL of enzyme samples were added by triplicate in 96-well thermocycler plates. The gradient scale from 30°C to 80°C was established as follows: 30,0°C, 31.6°C, 34.6°C, 39,5°C, 45,3°C, 49,6°C, 52,8°C, 55,0°C, 56,9°C, 59,9°C, 64,3°C, 69,7°C, 75,0°C, 78,1°C, 80,0°C. A thermo cycler was used with a set incubation time of 10 min for the temperature gradient, followed by 10 min at 4°C and 5 min at 20°C. Samples were then subjected to the ABTS assay described previously for the screening. The ratio between the residual activities at different incubation temperatures and the initial activity at room temperature were used to calculate the thermostability values. The *T*_50_ value was determined by the transition midpoint of the inactivation curve of the protein as a function of temperature, which in our case was defined as the temperature at which the enzyme lost 50% of its activity following an incubation of 10 min. Appropriate dilutions of enzyme samples were prepared in such a way that aliquots of 20 μL gave rise to a linear response in kinetic mode.

### DNA Sequencing

Mutants were sequenced by GATC-Eurofins Genomics (Germany). Samples contained 5 µL of plasmid (100 ng/μL) and 5 µL of the primer (5 µM). Four primers (RMLN, RMLC, apo1secdir, apo1secrev) were used separately for each mutant in order to cover the whole sequence.

## Results and Discussion

**FIGURE 2:**
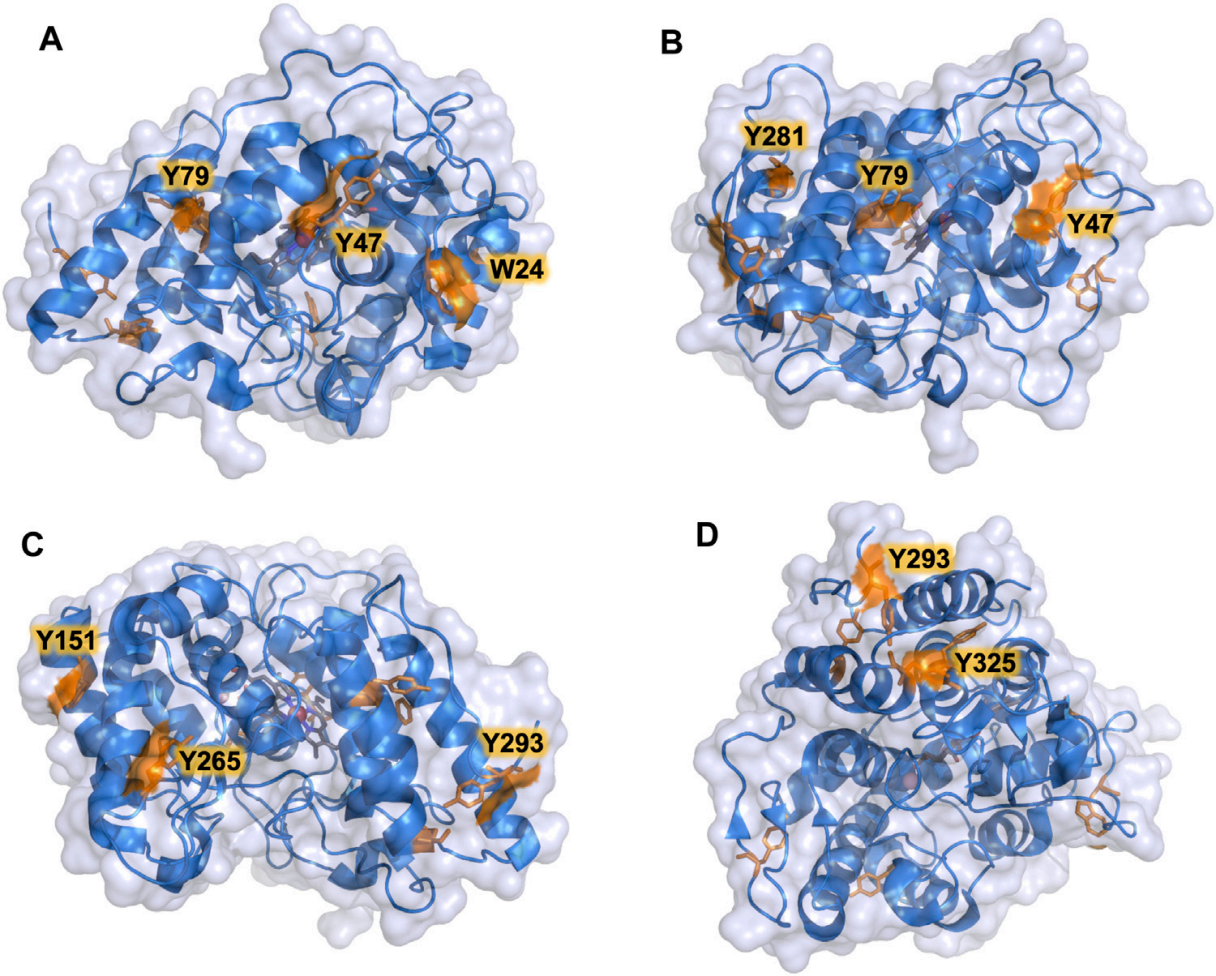
Surface exposed Trp and Tyr residues in PaDa-I variant. Residues are highlighted in orange. **(A)** Trp24, Tyr47, Tyr79. **(B)** Tyr47, Tyr79, Tyr281. **(C)** Tyr151, Tyr265, Tyr293. **(D)** Tyr293, Tyr325. Models were generated using the PyMOL Molecular Graphics System (version 2.4.2 Schr dinger, LLC) and based on the crystal structure of the PaDa-I mutant at a resolution of 1.5 Å (PDB: 5OXU).

**FIGURE 3:**
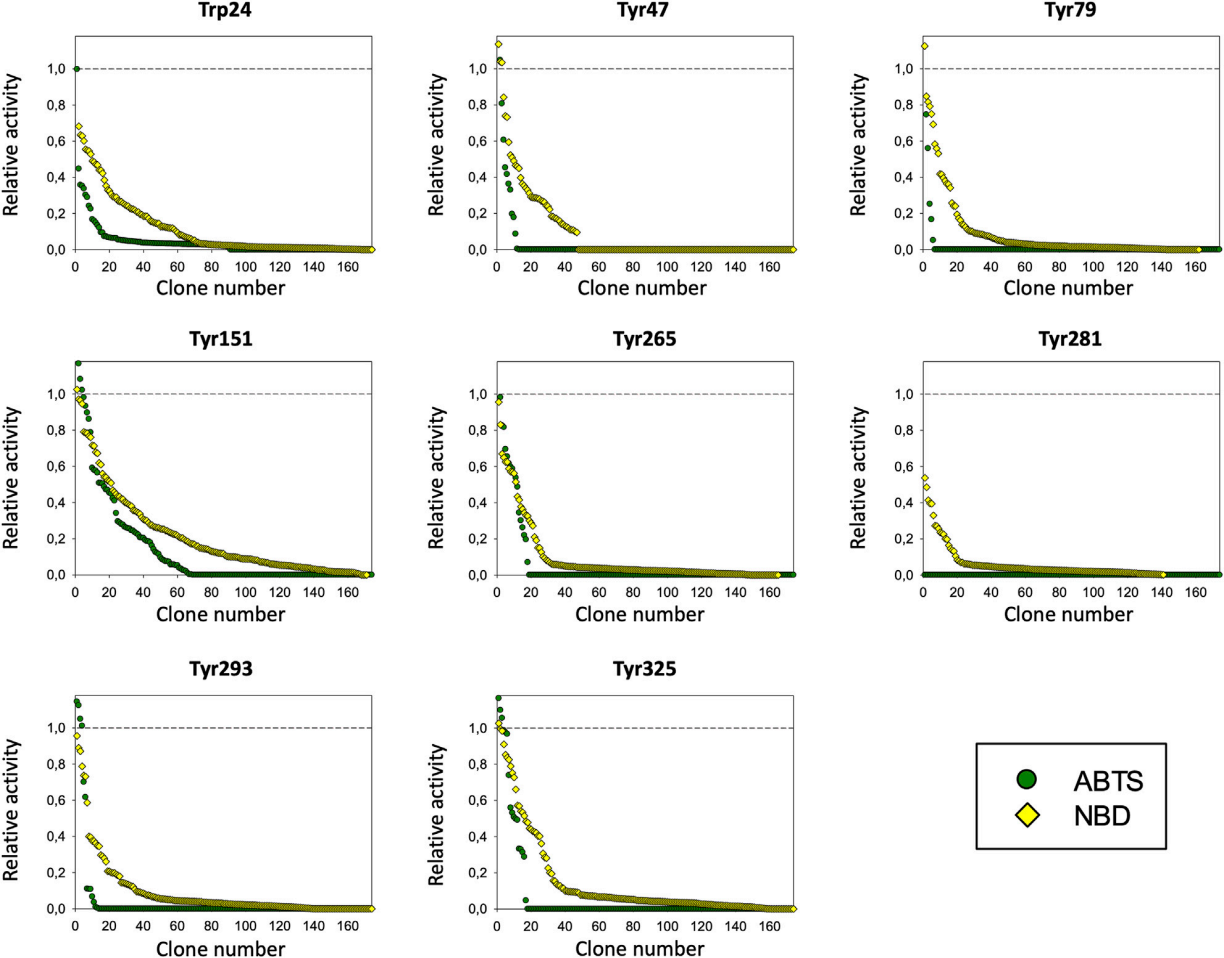
Mutagenic landscapes of the saturation mutagenesis libraries of the surface Trp and Tyr residues. The activity of the clones is plotted in descending order and the horizontal dashed line indicates the activity of the parental PaDa-I variant.

The PaDa-I variant represented the starting point for this study, a secretion mutant of *Aae*UPO that was generated after five rounds of random mutation and *in vivo* DNA recombination by directed evolution (Molina-Espeja et al., 2014). The PaDa-I mutant carries four mutations in the signal peptide (F12Y, A14V, R15G and A21D) and five mutations in the mature protein (V57A, L67F, V75I, I248V and F311L), which together drive strong expression in yeast (8 mg/L in *Saccharomyces cerevisiae* and over 200 mg/L in *Pichia pastoris*), coupled to good stability and high activity (Molina-Espeja et al., 2015). To rank which oxidizable aromatic residues might be responsible for the peroxidative activity of this enzyme, we firstly performed mixed QM/MM simulations using Qsite in pH 4.5 (for peroxidative activity) and 7.0 (for peroxygenative activity). The analysis focused on all the tyrosine (Tyr47, Tyr79, Tyr151, Tyr160, Tyr194, Tyr265, Tyr281, Tyr293 and Tyr325) and tryptophan (Trp24) residues in the quantum region, subtracting one electron and computing the spin density. QM/MM results at pH 7.0 suggested that Trp24 (62%) and Tyr47 (38%) have more tendency to be oxidized over other residues, Supplementary Figure 1. On the other hand, spin density at pH 4.5 was distributed among Trp24 (47%), Tyr47 (4%), Tyr151 (33%) and Tyr160 (13%). These calculations served as a ranking of the preferential oxidation site, but they did not address the actual existence of a distant oxidation through LRET. At this point, we constructed independent saturation mutagenesis libraries of the selected Trp24 and surface Tyr in PaDa-I (Tyr47, Tyr79, Tyr151, Tyr265, Tyr281, Tyr293 and Tyr325), Figure 2. We excluded Tyr160 and Tyr194 from these experiments as their side chains are not directly exposed to the solvent. The eight mutant libraries were screened using colorimetric assays for peroxidative activity (ABTS) and peroxygenative activity (NBD), Figure 3. The mutational landscapes of all the residues studied were very detrimental, as roughly 60–90% of the clones were inactive when measured with ABTS and 50–90% with NBD, Table 1. Nevertheless, after two consecutive re-screenings nine variants were selected, produced and preliminary characterized, Table 2. None of the mutants had significant differences in their two activities, although they were affected by the mutations in a similar manner. For instance, in terms of both activities and regardless of the substrate tested, the Trp24 that was considered the most oxidizable residue in the QM/MM analysis was very sensitive when mutated to Gly, and to a lesser extent when replaced by Tyr. This result agrees with our previous directed UPO evolution campaign for the synthesis of the agrochemical 1-naphthol, in which we designed the W24F mutant that had a similar decrease in both peroxygenative and peroxidative activities (Molina-Espeja et al., 2016). Although mutations in potential oxidizable residues seem to affect the peroxidative activity of UPOs, the decrease in the peroxygenative activity observed indicates that such residues are not directly connected to a LRET pathway as both activities are modified upon mutation to a greater or lesser extent. Instead, problems associated with correct folding and stability through the yeast’s secretory route may be responsible for these effects, consistent with the large number of inactive clones in the mutant libraries, Table 1 and Figure 3.

**TABLE 1:** Inactive clones^a^ (expressed as a %) per library and substrate.

Mutant library	ABTS	NBD
Trp24	92	66
Tyr47	94	74
Tyr79	97	84
Tyr151	72	48
Tyr265	90	84
Tyr281	100	90
Tyr293	95	80
Tyr325	91	77
Gly314-Gly318 loop	99	95

a We define an inactive clone as any variant of the mutant library with activity below 10% of the parental PaDa-I.

**TABLE 2:** Activity of selected clones from the saturation mutagenesis libraries on oxidizable aromatic surface residues.

Library	Clone	Nucleotide change	Amino acid change	ABTS^a^	NBD^a^
Trp24	2E5	_70_GGT_72_	W24G	0.27	0.01
Trp24	1F7	_70_TAT_72_	W24Y	0.61	0.33
Tyr47	1A5	_139_TGG_142_	Y47W	1.03	0.93
Tyr47	2D9	_139_GTC_142_	Y47V	0.22	0.09
Tyr79	2B11	_235_CTG_237_	Y79L	0.26	0.08
Tyr79	2H12	_235_ATC_237_	Y79I	0.33	0.24
Tyr265	1F9	_793_TGG_795_	Y265W	0.75	0.70
Tyr281	1C5	_841_CTG_843_	Y281L	0.29	0.02
Tyr293	1D11	_877_CCG_879_	Y293P	0.23	0.01

a Measured in triplicate on supernatants of independent cultures grown in 96-well plates. Activities are normalized and expressed relative to the parental PaDa-I.

While the true location of the oxidation site for the peroxidative activity in UPOs remains elusive, the peroxygenative oxidation site has been well-defined (Hofrichter and Ullrich, 2014). This activity allows UPOs to introduce oxygen functionalities into organic compounds stemming from Compound I, a reactive oxo ferryl cation radical complex (^+•^Heme-Fe^4+^ = O) that follows a peroxide shunt pathway. The oxygen transfer reaction takes place within the heme cavity, such that the substrate is positioned at a Van der Waals distance from Compound I with the help of an inner triad of Phe residues that orientates the substrate towards the heme of the PaDa-I variant (Ramirez-Escudero et al., 2018). Interestingly, we identified a flexible Gly314-Gly318 loop at the entrance of the heme funnel that adopts different conformational states, and that may affect the diffusion of substrates and products, altering their residence time at the heme access channel (Ramirez-Escudero et al., 2018). Indeed, the plasticity of this loop was studied previously, subjecting Ala316 to saturation mutagenesis and generating the JEd-I variant that carries an A316P substitution that notably modifies both peroxidative and peroxygenative activities. This finding is in good agreement with other mutations around the heme access channel previously introduced by laboratory evolution, suggesting that the heme access channel may be responsible for the peroxidative activity of UPOs (Molina-Espeja et al., 2016; Gomez de Santos et al., 2018).

**FIGURE 4:**
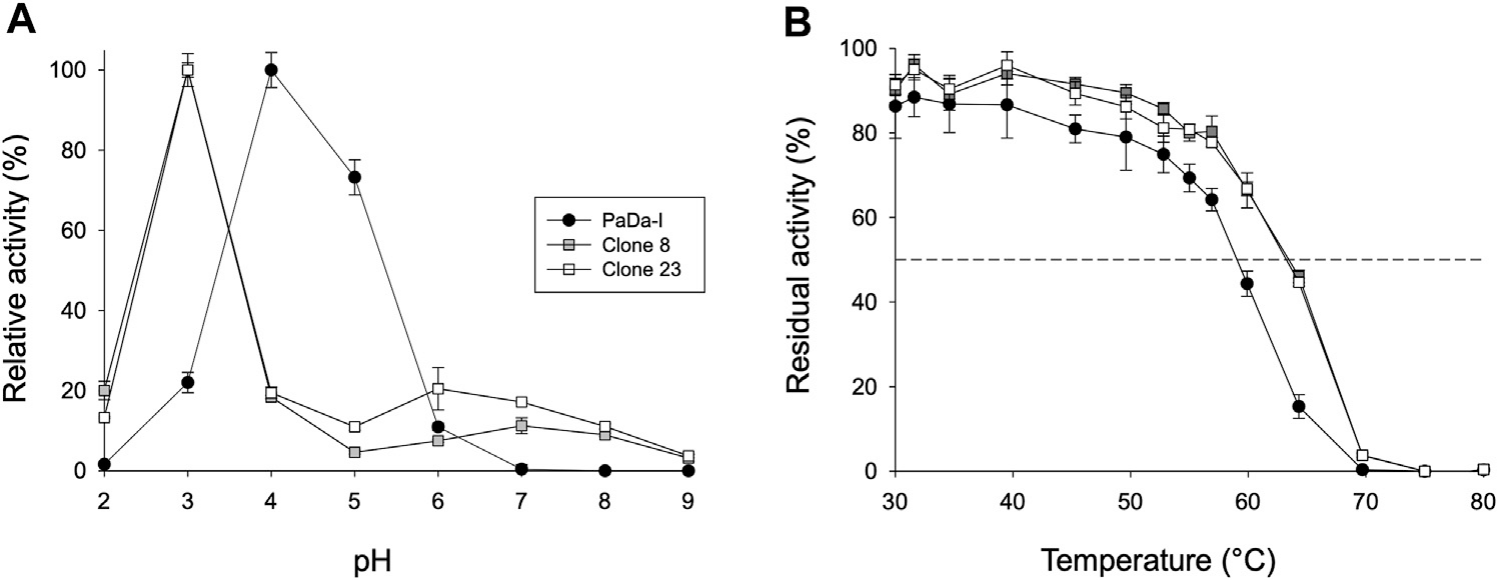
Biochemical characterization. **(A)** pH activity profile. Activities were measured at different pHs in 100 mM citrate/phosphate/borate buffer with 2 mM H_2_O_2_ and 2 mM ABTS. The activity value was normalized to the optimum activity, and each point represents the mean and standard deviation of three independent experiments. **(B)** Thermostability (*T*_50_). Each point represents the mean and standard deviation of three independent experiments: PaDa-I (black circles), clone 8 (grey squares), and clone 23 (white squares).

To assess whether or not the peroxidative activity in PaDa-I could be controlled by residues in this region, we performed a combinatorial saturation mutagenesis (CSM) experiment on the five amino acids that make up the Gly314-Gly318 loop. The _314_GVAAG_318_ segment was amplified by high-fidelity PCR using NDT degenerate codons to saturate each residue with the 12 characteristic amino acids (Gly, Phe, Ile, Leu, Val, Tyr, His, Cys, Ser, Asn, Asp, Arg), covering the main biophysical characteristics of amino acids. Given the absence of ultra-high-throughput screening methods available for UPOs, a full exploration of this library was not performed but rather, only a small yet representative fraction was examined. We roughly screened 1,400 clones with ABTS and NBD as substrates. Regardless of the substrate, the CSM landscapes were again highly deleterious (over 95% of inactive clones), indicating the sensitivity of this loop to mutation, Table 1. Although only a small fraction of the possible mutants was covered and characterized biochemically by the screening, two functional variants were identified (clones 8 and 23), purified and characterized biochemically, Supplementary Figure 2. We observed a dramatic shift in the optimal pH for ABTS, from 4.0 to 5.0 with the Pada-I variant to 3.0 with clones 8 and 23, Figure 4A, an indication that peroxidative activity could be located in the heme access channel. In terms of kinetic stability, clones 8 and 23 were functional and very stable, with an increase in the *T*_50_ (the temperature at which the enzyme retains 50% of its activity after a 10 min incubation) of roughly 4.5°C, Figure 4B. Steady kinetic parameters were determined for the peroxidative ABTS, and for the peroxygenative substrates NBD and veratryl alcohol, Table 3 **,** Supplementary Figure 3. Both clones 8 and 23 conserved similar kinetic parameters as the PaDa-I for the peroxygenative substrates, whereas their catalytic efficiency for ABTS increased 2-fold relative to that of PaDa-I. Although the *k*_cat_ for ABTS diminished ∼1.6-fold, the *K*_m_ decreased roughly 3.5-fold. Consequently, we confirmed that mutations in the Gly314-Gly318 loop of the heme access channel notably affected the kinetic parameters, as well as the pH activity profile, for ABTS, evidence that this region is the true origin for the peroxidative activity of the PaDa-I UPO variant. By contrast, and in the light of the saturation mutagenesis of the Tyr/Trp surface residue libraries, LRET pathways are ruled out as the potential origin of peroxidative activity.

**TABLE 3:** Steady kinetic parameters.

Substrate	Kinetic constants	PaDa-I	Clone 8	Clone 23
ABTS	*K*_m_ (mM)	0.048 ± 0.04	0.014 ± 0.0024	0.015 ± 0.002
	*k*_cat_ (s^−1^)	395 ± 13	239 ± 13	250 ± 11
	*k*_cat_/*K*_m_ (s^−1^ mM^−1^)	8200 ± 600	16830 ± 3027	16667 ± 2340
NBD	*K*_m_ (mM)	0.483 ± 0.095	0.437 ± 0.053	0.376 ± 0.040
	*k*_cat_ (s^−1^)	338 ± 22	368 ± 19	367 ± 16
	*k*_cat_/*K*_m_ (s^−1^ mM^−1^)	700 ± 99	842 ± 111	976 ± 112
Veratryl Alcohol	*K*_m_ (mM)	6.20 ± 0.7	7.90 ± 0,82	4.92 ± 0.45
	*k*_cat_ (s^−1^)	121 ± 5	183 ± 7	151 ± 4
	*k*_cat_/*K*_m_ (s^−1^ mM^−1^)	19 ± 1	23 ± 3	31 ± 3

Kinetic constants were measured at optimum pH values for each substrate: pH 3.0 (clones 8 and 23) and pH 4.0 (PaDa-I) for ABTS; pH 7.0 (clones 8, 23 and the PaDa-I) for NBD and veratryl alcohol.

**FIGURE 5:**
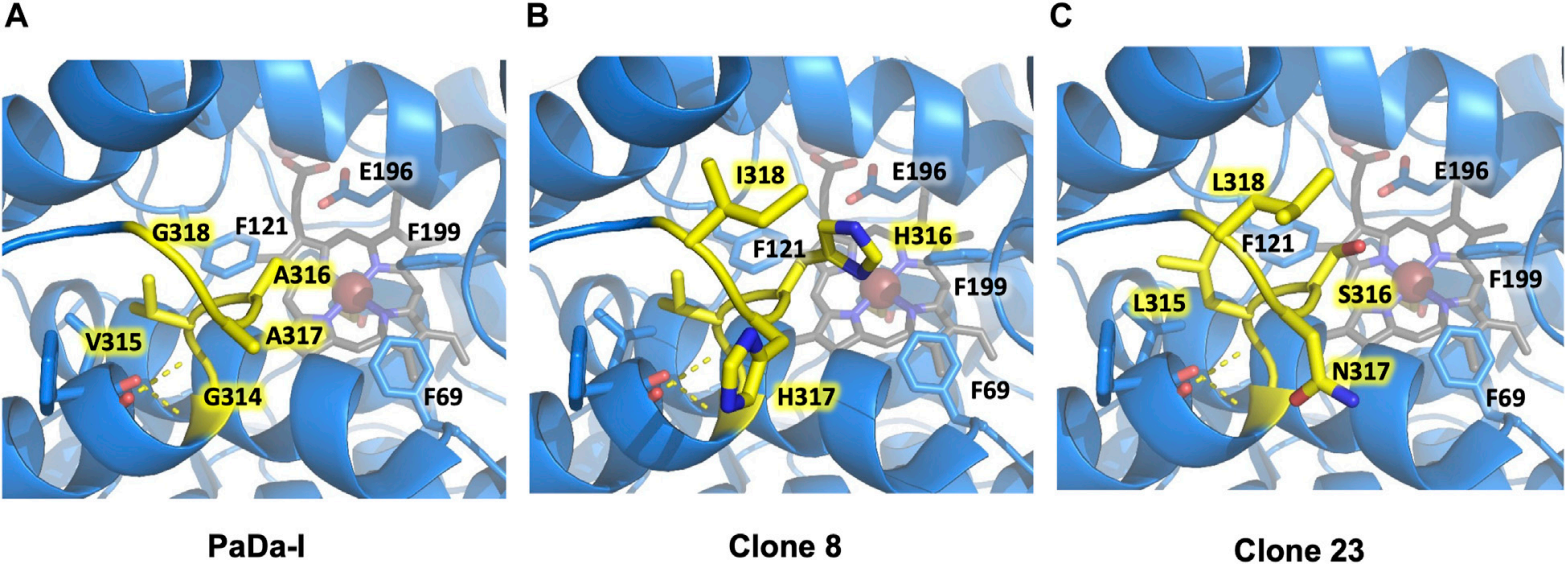
Flexible loop adjacent to the heme-access channel. Residues forming the loop are highlighted in yellow. **(A)** PaDa-I. **(B)** Clone 8. **(C)** Clone 23. Only mutated residues in the flexible loop are highlighted for clones 8 and 23. Other relevant amino acids for A*ae*UPO catalysis (Phe69, Phe121, Phe199, Glu196) are also indicated. Models were generated using the PyMOL Molecular Graphics System (version 2.4.2 Schr dinger, LLC) and based on the crystal structure of the PaDa-I mutant at a resolution of 1.5 Å (PDB: 5OXU).

**FIGURE 6:**
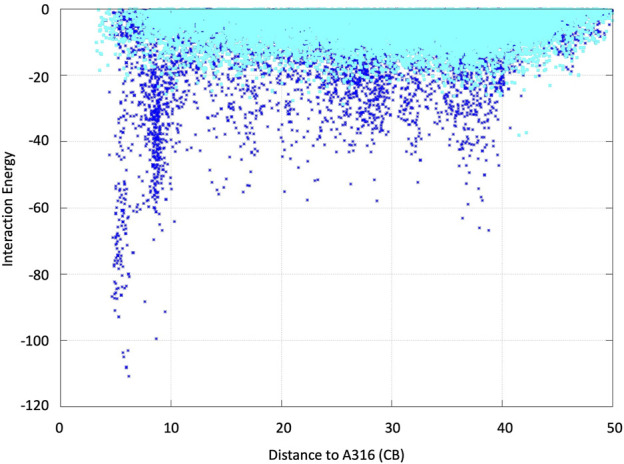
ABTS interaction energy profile against the distance of Ala316s beta carbon along the diffusion simulations with PELE. Both the doubly negative (dark blue) and the neutral (cyan) substrate species were assayed. Interaction energies are shown in kcal/mol and distances in angstroms.

**FIGURE 7:**
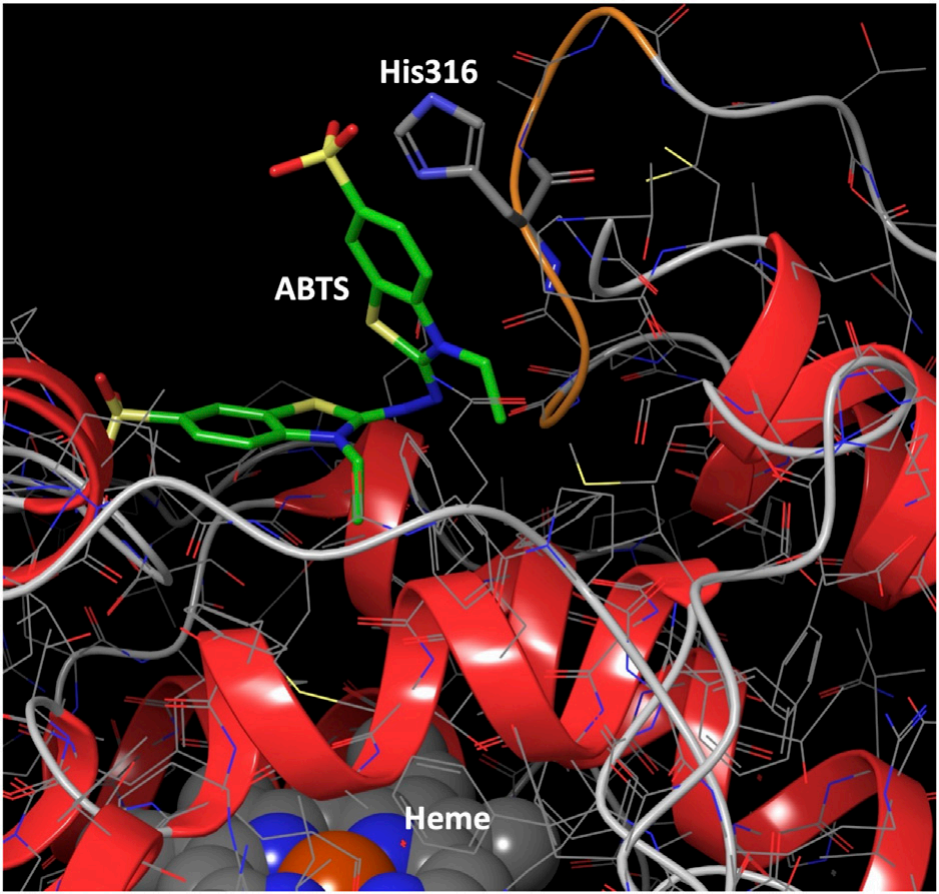
Binding mode for ABTS best interaction energy pose shown in Figure 5. ABTS (in tube representation with green carbons), the heme cofactor (spacefill representation at the **bottom**) and residue 316 (in tube representation) are shown. The loop ribbon of residues 314–318 is also highlighted in orange. Just for illustration, from the best interaction energy pose, residue 316 has been mutated to a protonated histidine and optimized while keeping the rest frozen. All hydrogens but those of His316 have been omitted for clarity.

We analyzed the mutations in clone 8 (A316H-A317H-G318I) and clone 23 (V315L-A316S-A317N-G318L), Figure 5. The loop that shapes the heme funnel is very flexible, with different conformations observed in the crystal complexes (Ramirez-Escudero et al., 2018). Thus, it was somewhat surprising that after introducing three to four consecutive mutations in the loop the variants were still functional and much more stable than the parental type PaDa-I. To further address the oxidation of ABTS at the entrance channel, we performed substrate diffusion simulation studies with PELE algorithm [Protein Energy Landscape Exploration, (Lecina et al., 2017)]. For this, we used the binding pocket protocol, which probes the entire enzyme surface without any bias. Figure 6 shows the exploration results when using both ABTS protonation states: the doubly negatively charged or the neutral (protonation at the mid nitrogen) species. Clearly, while the neutral species introduces a well-defined minima (dark blue color in Figure 6), the negative substrate does not show any apparent binding pocket in the surface (cyan color in Figure 6); the dominance of the neutral species seems to agree with the optimal pH and with previous studies (Delavari and Perez, 2013). Importantly, the binding minima is located at ∼5Å from Ala316s beta carbon, presenting a significant penetration into the active site entrance pocket, Figure 7. Based on this complex model, the role of Gly314-Gly318 in stabilizing ABTS seems quite possible. In a quick calculation, for example, we mutated Ala316 into a (protonated) His, as in clone 8, and the docking score of ABTS increased one unit when using the Glide SP scoring function. While only further crystallographic soaking experiments of the variants will clarify the exact influence of these mutations, it seems reasonable to think that they are involved in the access and/or stabilization of peroxidative substrates to the channel.

## Conclusion

UPO is an attractive biocatalyst capable of performing a wide array of complex oxygen-transfer reactions due to its peroxygenative activity, although the convergence of this with peroxidative activity still represents a concern when it is considered for use in organic synthesis. Here, we identified true catalytic determinants of peroxidative activity located at the heme access channel, while ruling out any potential LRET route from the protein surface to the heme. In the near future, we will attempt to engineer UPO variants in which peroxidative activity is quenched while their peroxygenative activity for any desired biotransformation is enhanced.

## Data Availability

The original contributions presented in the study are included in the article/Supplementary Material, further inquiries can be directed to the corresponding author.
