# Corrigendum: Mapping Potential Determinants of Peroxidative Activity in an Evolved Fungal Peroxygenase From *Agrocybe aegerita*

**DOI:** 10.3389/fbioe.2021.778727

**Published:** 2021-10-05

**Authors:** Patricia Molina-Espeja, Alejandro Beltran-Nogal, Maria Alejandra Alfuzzi, Victor Guallar, Miguel Alcalde

**Affiliations:** ^1^ Department of Biocatalysis, Institute of Catalysis, CSIC, Madrid, Spain; ^2^ Barcelona Supercomputing Center, Barcelona, Spain; ^3^ ICREA, Institució Catalana de Recerca i Estudis Avançats Passeig Lluís Companys, Barcelona, Spain

**Keywords:** fungal unspecific peroxygenase, peroxidative activity, peroxygenative activity, long range electron transfer pathway, heme access channel, directed evolution

In the original article, there was a typo error in the **Introduction** section, page 3, second paragraph, in which high and low efficiency were interchanged inaccurately. The sentence should be corrected as follows:

“Conversely, the heme access channel has been long described as the main oxidation site for low-redox potential compounds in generic peroxidases, although VP oxidizes low-redox potential compounds at both the heme access channel (with low efficiency) and at the catalytic surface Trp [with high efficiency (Ruiz-Dueñas et al., 2008)].”

The authors apologize for this error and state that this does not change the scientific conclusions of the article in any way. The original article has been updated.

