# Supplementary material for: Mapping Potential Determinants of Peroxidative Activity in an Evolved Fungal Peroxygenase from Agrocybe aegerita
Source: Front Bioeng Biotechnol. 2021 Sep 14;9:741282. doi: 10.3389/fbioe.2021.741282 (PMC8476742; doi:10.3389/fbioe.2021.741282)
Supplement: Supplementary file 1 [file DataSheet1.PDF]

SUPPLEMENTARY MATERIAL FOR:

Mapping potential determinants of peroxidative activity in an evolved fungal peroxygenase from *Agrocybe aegerita*

Patricia Molina-Espeja[1], Maria Alejandra Alfuzzi[1], Alejandro Beltran-Nogal[1], Victor Guallar[2,3] and Miguel Alcalde[1]

[1] Department of Biocatalysis, Institute of Catalysis, CSIC, Cantoblanco, Madrid, Spain.

[2]Barcelona Supercomputing Center, Barcelona, Spain.

[3]ICREA: Institució Catalana de Recerca i Estudis Avançats Passeig Lluís Companys, Barcelona, Spain

*Corresponding author: malcalde@icp.csic.es

Running title: Determinants of peroxidative activity in fungal peroxygenases.

Keywords: fungal unspecific peroxygenase, peroxidative activity, peroxygenative activity, long range electron transfer pathway, heme access channel, directed evolution.

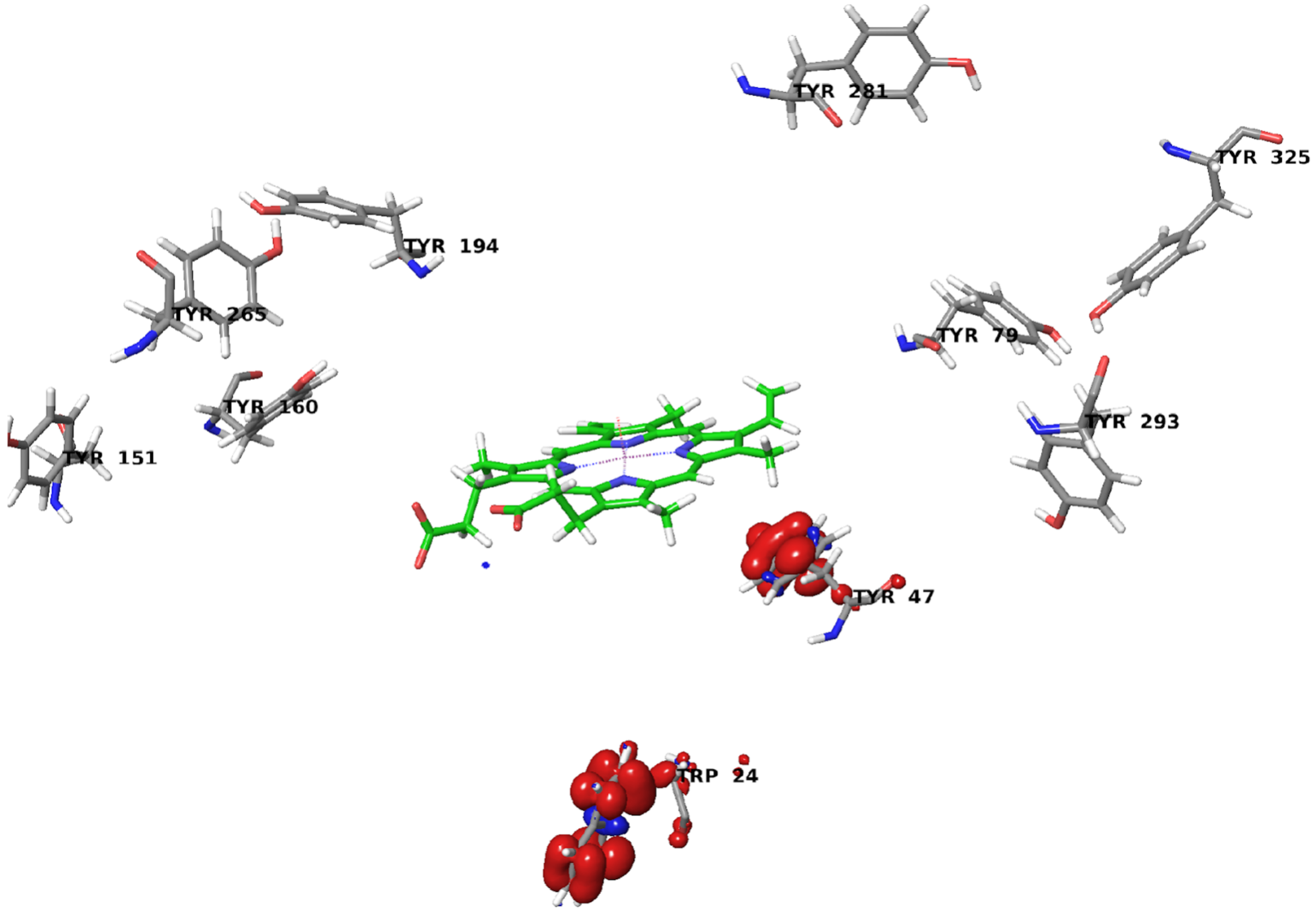

Figure S1. QM region used for the QM/MM calculations. The figure also depicts the total spin density at pH 7.0, mostly residing at Trp24 and Tyr47.

A B

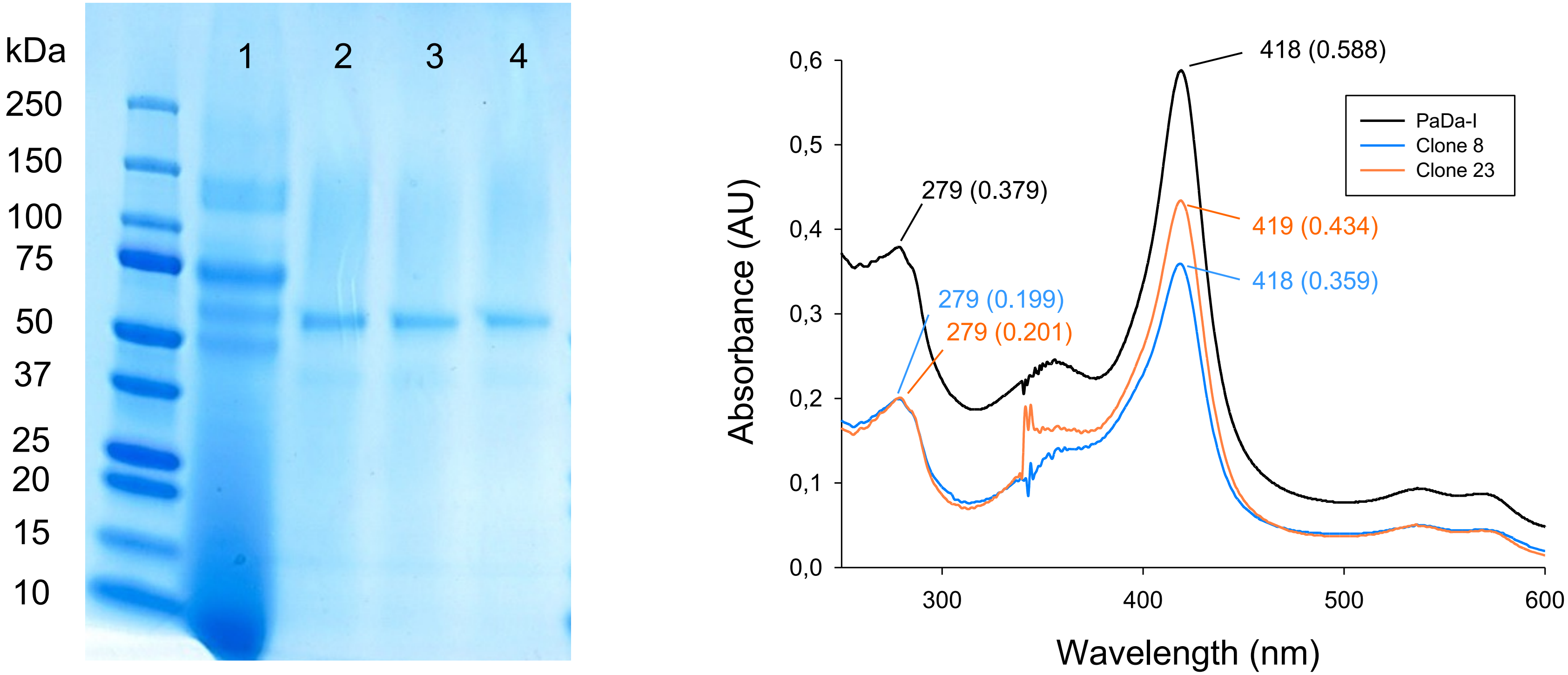

C

UPO	Rz (A_{418}/A_{280})
PaDa-I	1,55
Clone 8	1,80
Clone 23	2,16

Figure S2. Molecular mass and purification of UPOs. (**A**) SDS-PAGE analysis of purified fractions. Lines: (**1**) Concentrated supernatant, (**2**) PaDa-I, (**3**) clone 8, (**4**) clone 23. (**B**) UV-Vis spectra of purified PaDa-I (black line), clone 8 (blue line) and clone 23 (orange line). Absorption maxima values around 280 nm and 418 nm are displayed within the graphic. (**C**) Reinheitszahl values (Rz, A_{418}/A_{280}) of purified fractions of each UPO variant.

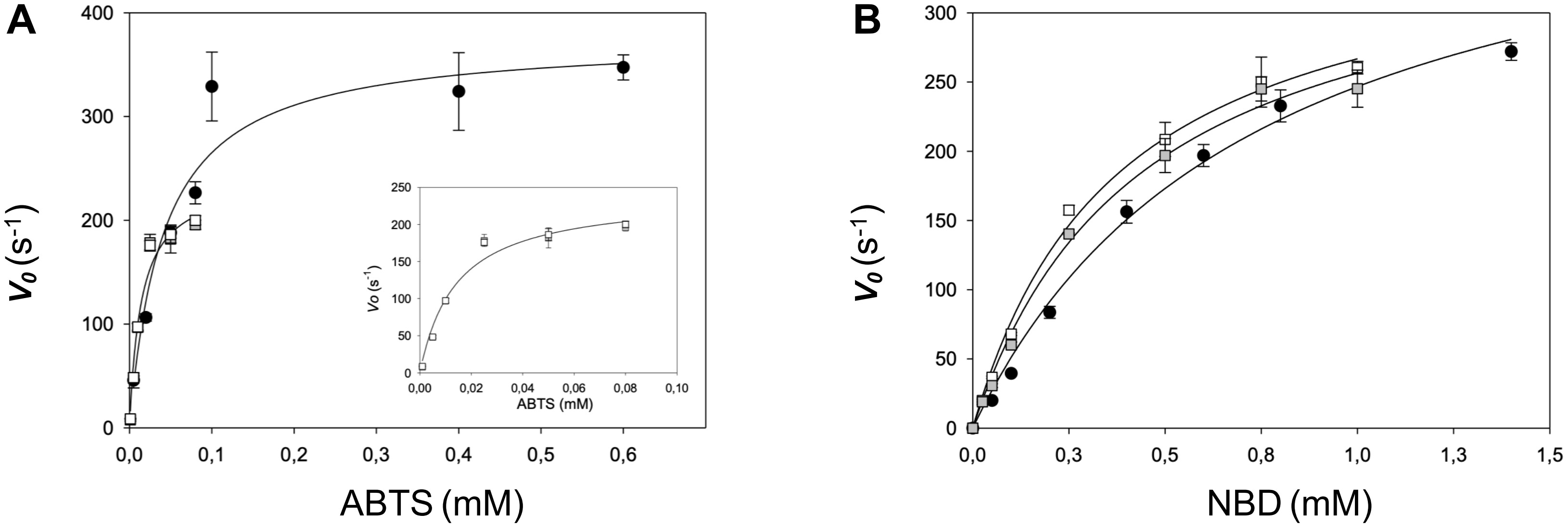

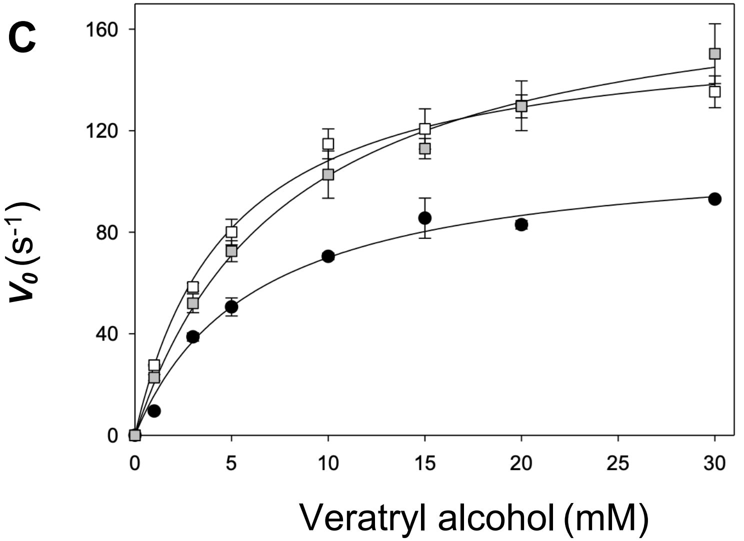

D

Substrate	PaDa-I	Clone 8	Clone 23
ABTS	4.5×10^{-4}	8.7×10^{-4}	9.3×10^{-4}
NBD	2.8×10^{-3}	8.7×10^{-3}	9.3×10^{-3}
Veratryl alcohol	9.0×10^{-3}	8.8×10^{-3}	9.3×10^{-3}

Figure S3. Kinetic measurements of parental type PaDa-I and clones 8 and 23 with peroxidative and peroxygenative substrates. (**A**) ABTS (inset showing the Michaelis-Menten plot of clones 8 and 23 in a more appropriate scale). (**B**) NBD. (**C**) Veratryl alcohol. Each point represents the mean and standard deviation of three independent experiments: PaDa-I (black circles), clone 8 (grey squares), and clone 23 (white squares). (**D**) Enzyme concentration (in µM) used in kinetic measurements.

Table S1. Primers used in this study.

RMLN	5’-CCTCTATACTTTAACGTCAAGG-3’
RMLC	5’- GGGAGGGCGTGAATGTAAGC-3’
apo1secdir	5’-GAAGGCGACGCCAGTATGACC-3’
apo1secrev	5’-GGTCATACTGGCGTCGCCTTC-3’
DIRSAT-W24	5’-ATACTCTGGCATCTCACGGGNDTCTCCCGAGAAATGGCGTTG-3’
REVSAT-W24	5’-GCCTCGTCGTTCACCAACTTTGCAGAGCTATTCTCGAGAGG-3’
DIRSAT-Y47	5’-ATACTCTGGCATCTCACGGGNNSCTCCCGAGAAATGGCGTTG-3’
REVSAT-Y47	5’-CAACGCCATTTCTCGGGAGSNNCCCGTGAGATGCCAGAGTAT-3’
DIRSAT-Y79	5’-CAAGCCGCAATCTTCGCCACANNSGCGGCCCACCTTGTGGACG-3’
REVSAT-Y79	5’-CGTCCACAAGGTGGGCCGCSNNTGTGGCGAAGATTGCGGCTTG-3’
DIRSAT-Y151	5’-CTTCGAACAGTTGGTTGACNNSAGCAACCGATTTGGAGGAG-3’
REVSAT-Y151	5’-CTCCTCCAAATCGGTTGCTSNNGTCAACCAACTGTTCGAAG-3’
DIRSAT-Y265	5’-GTCGGCAAGATCAACAGCNNSACCGTCGACCCAACATCCTCTG-3’
REVSAT-Y265	5’-CAGAGGATGTTGGGTCGACGGTSNNGCTGTTGATCTTGCCGAC-3’
DIRSAT-Y281	5’-CTTTTCCACCCCCTGCTTGATGNNSGAGAAATTCGTCAACATAAC-3’
REVSAT-Y281	5’-GTTATGTTGACGAATTTCTCSNNCATCAAGCAGGGGGTGGAAAAG-3’
DIRSAT-Y293	5’-CATAACGGTCAAGTCACTCNNSCCGAATCCGACGGTG-3’
REVSAT-Y293	5’-CACCGTCGGATTCGGSNNGAGTGACTTGACCGTTATG-3’
DIRSAT-Y325	5’-GATGTACCCAGGTCTTCCCANNSGGGCGAGATTGAGCG-3’
REVSAT-Y325	5’-CGCTCAATCTCGCCCSNNTGGGAAGACCTGGGTACATC-3’
DIRSAT-CSM	5’-AATCTCGATTTCTTATTCCAGNDTNDTNDTNDTNDTTGTACCCAGGTCTTCCCATACGGGCGA-3’
REVSAT-CSM	5’TCGCCCGTATGGGAAGACCTGGGTACAAHNAHNAHNAHNAHNCTGGAATAAGAAATCGAGATT-3’
